# Nanoplasmonic Structure of a Polycarbonate Substrate Integrated with Parallel Microchannels for Label-Free Multiplex Detection

**DOI:** 10.3390/polym13193294

**Published:** 2021-09-27

**Authors:** Yi-Jung Lu, Han-Yun Hsieh, Wen-Chang Kuo, Pei-Kuen Wei, Horn-Jiunn Sheen, Hidetoshi Tahara, Te-Wei Chiu, Yu-Jui Fan

**Affiliations:** 1 Division of Family and Operative Dentistry, Department of Dentistry, Taipei Medical University Hospital, Taipei 11031, Taiwan; 893031@h.tmu.edu.tw; 2 Institute of Applied Mechanics, National Taiwan University, Taipei 10617, Taiwan; h_yun@tmu.edu.tw (H.-Y.H.); oscar9320@gmail.com (W.-C.K.); sheenh@ntu.edu.tw (H.-J.S.); 3 Department of Cellular and Molecular Biology, Graduate School of Biomedical Sciences, Hiroshima University, Hiroshima 734-8553, Japan; toshi@hiroshima-u.ac.jp; 4 School of Biomedical Engineering, Taipei Medical University, Taipei 11031, Taiwan; 5 Research Center for Applied Sciences, Academia Sinica, Nankang, Taipei 11529, Taiwan; pkwei@sinica.edu.tw; 6 Department of Materials and Mineral Resources Engineering, National Taipei University of Technology, Taipei 10608, Taiwan

**Keywords:** surface plasmon resonance (SPR), biosensor, nanoimprinting, multiplex detection

## Abstract

In this study, a multiplex detection system was proposed by integrating a localized surface plasmon resonance (LSPR) sensing array and parallel microfluidic channels. The LSPR sensing array was fabricated by nanoimprinting and gold sputter on a polycarbonate (PC) substrate. The polydimethylsiloxane (PDMS) microfluidic channels and PC LSPR sensing array were bound together through (3-aminopropyl)triethoxysilane (APTES) surface treatment and oxygen plasma treatment. The resonant spectrum of the LSPR sensing device was obtained by broadband white-light illumination and polarized wavelength measurements with a spectrometer. The sensitivity of the LSPR sensing device was measured using various ratios of glycerol to water solutions with different refractive indices. Multiplex detection was demonstrated using human immunoglobulin G (IgG), IgA, and IgM. The anti-IgG, anti-IgA, and anti-IgM were separately modified in each sensing region. Various concentrations of human IgG, IgA, and IgM were prepared to prove the concept that the parallel sensing device can be used to detect different targets.

## 1. Introduction

Label-free biosensors generally use a transducer to convert biological signals, received from a recognition element, into readable information. The transducer is the critical element for determining the sensitivity, sensing range, and detection limit of the biosensors. In the past, several transducers for label-free immunosensing were developed, including quartz crystal microbalances (QCMs) [1,2,3], amperometric transducers [4,5,6,7], cantilever beams [8,9,10], electrochemical impedance spectroscopy (EIS) biosensors [11,12,13,14], preconcentrated immunosensing [15,16,17], surface plasmon resonance (SPR) [18,19,20], and nanobead Brownian motion [21,22,23]. Transducer fabrication of most current technologies is expensive, and they are not easy to mass-produce for rapid screening.

SPR sensors have been widely used in medical diagnostics, environmental monitoring, and food safety [24,25,26,27,28]. Conventional reflective-type SPR platforms utilize a prism-coupled gold thin film on a glass substrate to induce an evanescent wave as the resonant wavelength that can be changed based on the biomolecular binding affinity.

Recently, transparent-type SPR sensors were proposed by propagating normal incident light into period gold nanohole arrays or nanoslits to generate SPR for biosensing applications [29,30,31]. Compared to the reflective-type SPR, transparent-type SPR sensors benefit from having a small detection volume that shows the capability to integrate with multiplex microfluidic devices [18]. They also provide a feasible way to achieve chip-based, high-throughput, label-free detection for modern DNA and protein microarrays. Moreover, state-of-the-art transparent-type SPR sensors can be mass-produced by automatic and large-area nanoimprinting.

A narrower-resonance line width allows higher sensitivity of SPR biosensors. A strategy to obtain sharp spectral resonance is to develop a distinctly asymmetrically shaped spectrum, called Fano resonances. Fano-type spectra arise from overlapping between a broadband resonance and a narrow discrete resonance [32,33,34]. Recently, a polymer-based nanoslit capped with gold film was developed that can generate Fano resonance spectra in a transverse magnetic wave (TM)-polarized wave [35].

Microfluidic systems are widely used in chemical reaction procedures and biological applications such as sample separation [36,37,38], flow cytometry [39,40,41], and cell sorters [42,43,44]. Polydimethylsiloxane (PDMS) is often used for rapid-testing microfluidic designs. It can be fabricated as a microfluidic device by adding a curing agent and casting a prepared master mold. PDMS normally binds to PDMS or glass to become a microfluidic channel through plasma treatment, which limits applications of PDMS for binding another substrate.

**Figure 1:**
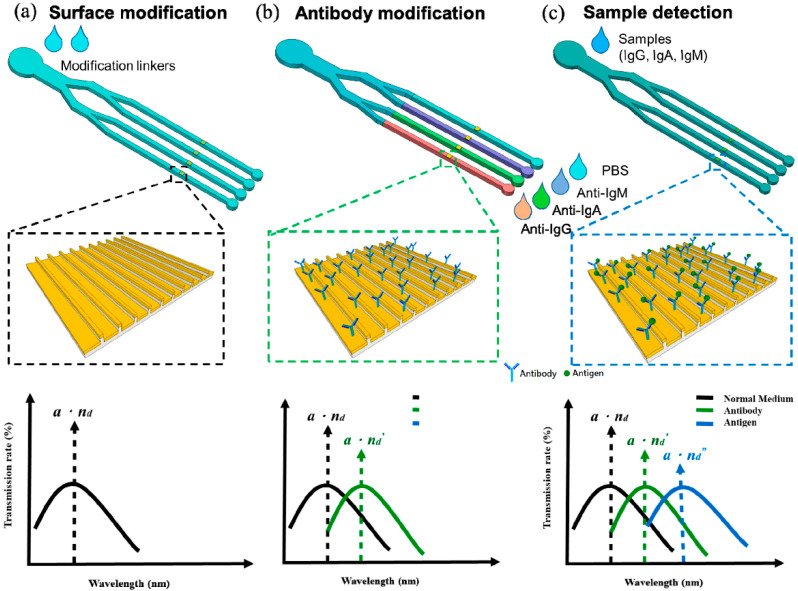
Sensing mechanism and operating procedures of the parallel microfluidic integrated nanoplasmonic sensing array for multiplex detection. (**a**) Surface modification. The cross linkers will flow into branches through main channel. The peak of the resonant wavelength is represented to *a*·n_d_. (**b**) Antibofy modification. To modifiy antibidies, the anti-IgA, -IgG, and -IgM will flow into sensing area through end of branches, separately. The peak of the resonant wavelength is represented to *a*·n_d_’, which is red-shift comparing to initial state (**c**).

In this study, we propose a parallel detection method through a nanoplasmonic sensing array integrated with parallel microfluidic channels that expands one major channel into four branching channels through two cascades. The nanoplasmonic sensing array can be mass-produced by automatically nanoimprinting on a polycarbonate (PC) substrate and sputter gold deposition. The PDMS-based microfluidic channel can be aligned and bound to a sensing array after chemical surface treatment and oxygen plasma treatment. The sensing mechanism is shown in Figure 1. The sensing regions were first activated by surface modification. Subsequently, anti-immunoglobulin G (IgG), anti-IgA, and anti-IgM were separately modified onto each sensing region as the probes. Because of refractive index changes at the sensing area’s surface with conjugated antibodies, the resonant spectra were red-shifted. For sensing analytes in samples, samples were loaded into the device to the sensing region. When the analytes were conjugated with antibodies, due to changes in the refractive index, the resonant spectra red-shifted again. The concentration of the analytes could be quantified by analyzing the red-shift of the peak values of the resonant spectra. The theoretical peak value of the resonant spectrum is given by

(1) $$\lambda =a\cdot n_{d}$$

where *a* is the period of nanostructure, and *n_d_* is the refractive index of the surface of the nanostructure. The innovation is that through microfluidic channel, multiplex LSPR sensing at the same time can be achieved. 

## 2. Materials and Methods

### 2.1. Device Fabrication

**Figure 2:**
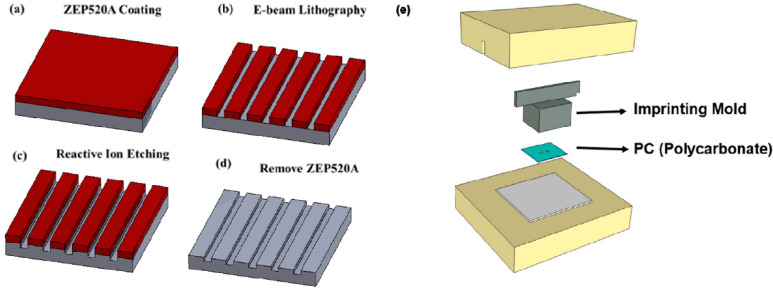
Fabrication of the mold for nanoimprinting. (**a**) spinning coating photoresist, (**b**) e-beam lithography to pattern photoresist, (**c**) RIE etching the silicon substrate, (**d**) remove photoresist and (**e**) use the silicon substrate as the mold to hot emboss PC substrate.

To fabricate the plasmonic nanostructure on the PC substrate, a nanoslit master was made. The photoresist, ZEP520A (ZEON Corp., Tokyo, Japan), was coated on the silicon wafer, and the straight line array, at 100 nm in width and with a period of 500 nm, was patterned by e-beam lithography. The silicon wafer was etched 50 nm in depth using a reactive ion etching (RIE, Oxford Instruments, Yatton, Bristol, UK) machine. After removing the photoresist, the master mold for nanoimprinting the structure on the PC substrate was obtained. The master mold consisted of 1×4 sensing regions, with each region having a total area of a 150-µm × 150-µm straight line array. The mold was used for nanoimprinting, and the pattern was transferred onto the PC substrate as Figure 2. Subsequently, a shield mask with openings corresponding to the 1 × 4 sensing regions was made using laser cut 3M tape (8003p, 3M, Harbor, MN, USA). Gold was then deposited at 60 nm thick by a DC sputter on the straight line array on the PC substrate covered with the shield mask. After removing the shield mask, the nanoplasmonic sensing array chip can be obtained.

The microfluidic system was designed as one inlet channel divided into four channels with four outlets through two cascades. The designed pattern was realized as an SU-8 mold on a silicon wafer with a thickness of 25 µm through photolithography. Polydimethylsiloxane (PDMS, Dow Corning, Denver, CO, USA ) was used to make the microfluidic channel. First, the PDMS was mixed with a curing agent at a 10: 1 volume ratio. After 30 min of degassing, the PDMS was poured into the SU-8 mold and left on a 65 °C hotplate for 2 h. After curing, the consolidated PDMS was peeled off from the mold, and inlet and outlet openings were made.

For the binding of PDMS and PC, PC was first surface-modified by (3-aminopropyl)triethoxysilane (APTES, Sigma Aldrich, St. Louis, MO, USA). Next, the PC substrate with a nanoplasmonic structure was immersed in a 1% *v/v* APTES water solution for 20 min. After washing with deionized (DI) water and drying with a nitrogen air stream, the APTES-modified PC and PDMS were treated with oxygen plasma at 18 W for 35 s. The oxygen plasma-treated PC and PDMS were then aligned, bound, and left on a 65 °C hotplate for 30 min.

### 2.2. Surface Modification

**Figure 3:**
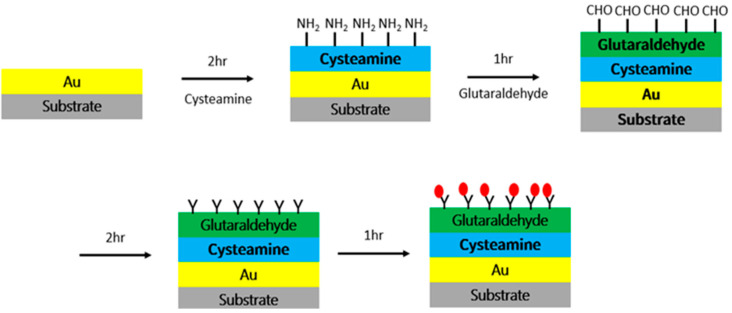
Process of antibody modification of the surface of the sensing device.

To detect IgG, IgM, and IgA in samples, the sensing regions of the device were first modified with secondary antibodies of IgG, IgM, and IgA separately on each sensing region. As shown in Figure 3, 100 µg/mL cysteamine was incubated in the sensing regions for 2 h, which allowed the cysteamine to bind to the gold surface via thiol groups, and the surface exhibited a net positive surface charge because of amine groups. After washing with phosphate-buffered saline (PBS), the sensing regions were incubated in 1 mM glutaraldehyde for 1 h, which a allowed the glutaraldehyde to bind to the amine groups of cysteamine. After glutaraldehyde modification, the devices were washed three times to remove any unbound glutaraldehyde. Subsequently, each sensing region was individually incubated with 1 mg/mL of a secondary antibody of IgG, IgM, or IgA for 2 h, after which the device was ready to use. For sensing, a mixture of IgG, IgM, and IgA samples were passed through the device to each sensing region for further detection.

### 2.3. Optical System

**Figure 4:**
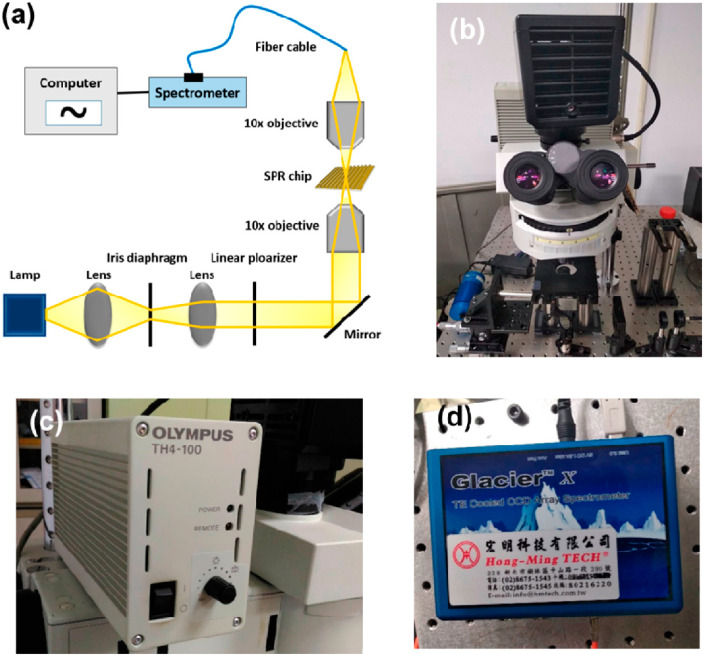
(**a**) Optical setup for a transparent type of nanoplasmonic biosensing device. (**b**) microscope (**c**) light source (**d**) spectrometer.

An optical setup for generating plasmon and measuring the resonant spectrum through the nanoplasmonic structure on the PC substrate was constructed as shown in Figure 4 Broadband white light at 100 W passed through the nanostructure chip through a 10× objective, and the optical signals were collected from the other side through another 10× objective. To filter out the resonant spectrum, a polarizer was used to extract resonant wavelengths in the transverse magnetic (TM) direction. The collected light signals were guided into a spectrometer through an optical fiber. The resonant spectrum was recorded, and the resonant spectrum shifted when the refractive index at the surface of the nanostructure changed due to protein conjugation, which could be used to estimate the sensitivity of the sensing chip.

## 3. Results and Discussion

### 3.1. Sensitivity Test

**Figure 5:**
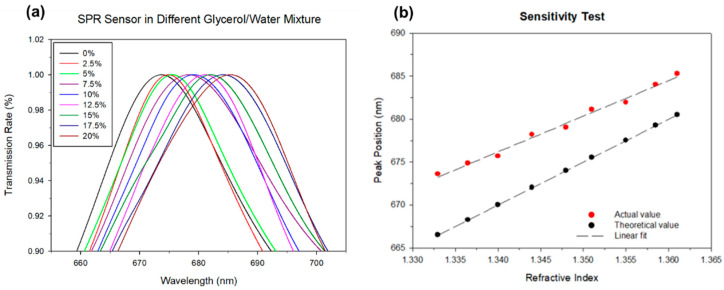
Sensitivity test. (**a**) Peak values of different volume ratios of glycerol to water solutions. (**b**) The peak value of the resonant spectrum versus the refractive index compared to theoretical values.

The sensitivity of the nanoplasmonic chip in the microfluidic channel was first investigated. Glycerol solutions with 0, 2.5, 5, 7.5, 10, 12.5, 15, 17.5 and 20 volume ratios to the total solution were prepared and passed through the microfluidic channels. The resonant spectra of these samples in the sensing region of the device were recorded and plotted in Figure 5a. Results indicated that the resonant spectra red-shifted due to an increase in the percentage of the glycerol solutions, which resulted in an increase in the refractive index. Subsequently, peak values of the resonant spectra versus the refractive index were plotted in Figure 5b.

The measured peak values of the resonant spectra were slightly larger than the theoretical ones, because the resonant spectrum was an asymmetric Fano-type resonant spectrum. Theoretical values were calculated as the mean of the peak and dip values of the Fano-type spectrum. In this experiment, peak values of the resonant spectra were measured so that the peak values differed from theoretical values.

### 3.2. Sample Test

The four branching channels were separately modified with anti-IgG, anti-IgA, and anti-IgM with concnetration of 500 μg/mL, with a bare channel as a reference. The modification processes are shown in Figure 3. The cysteamine and glutaraldehyde solutions were sequentially injected into the microfluidic channel through the inlet. After PBS washing, the anti-IgG, anti-IgA, and anti-IgM at a concentration of 1 mg/mL in PBS with pH of 7.4 were separately injected into each branch channel and incubated for 1 h. Thereafter, the resonant spectra of the sensing regions were recorded. For measuring wavelength shift, the temperature should be constant during measuring. We set at room temperature of 25 °C in this study. The red-shifts of the resonant wavepengthes in sensing area corresponding to IgA, IgG, and IgM were measured when anti-IgA, anti-IgG, and anti-IgM modified onto the sensing area respectively. It indicates that the antibodies were successfully modified onto their own sensing area. 

**Figure 6:**
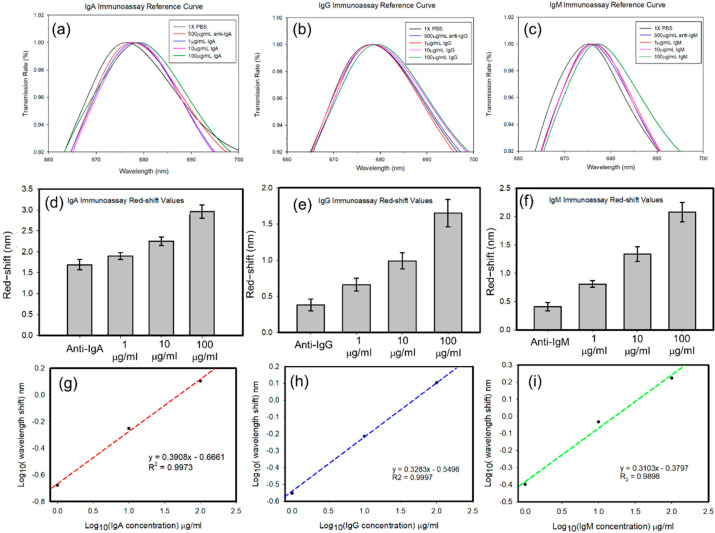
Resonant spectra of the different concentration of (**a**) immunoglobulin A (IgA), (**b**) IgG, and (**c**) IgM. Red-shift values of the different concentration of (**d**) IgA, (**e**) IgG, and (**f**) IgM. Linear regression of sensing results of (**g**) IgA, (**h**) IgG, and (**i**) IgM.

Sequentially, 1, 10, and 100 µg/mL of IgG, IgA, and IgM were pumped into the corresponding branch channels. The resonant spectra of the sensing regions were recorded. Results are revealed in Figure 6a–c, respectively. The results indicated that with increasing IgG, IgA, and IgM concentrations, the resonant spectra of the nanoplasmonic sensing chip red-shifted. The red-shift values were compared to peak values of resonant spectra with different sample concentrations with only the antibody. The red-shift values for sensing IgG, IgA, and IgM, were calculated and are respectively plotted in Figure 6d–f. Higher concentrations of the analytes caused greater red-shifts of the resonant spectra. This indicated that the developed nanoplasmonic sensing array integrated in parallel with a microfluidic device could be used for multiplex detection. We further plotted the averaged red-shifts value in logarithm versus various concentrations of IgA, IgG, and IgM in logarithm values, separately in Figure 6g–i. All of the results showed good linear ranges. The sensitivities of 0.3908, 0.3283, and 0.3103, for IgA, IgG, and IgM detection. 

**Figure 7:**
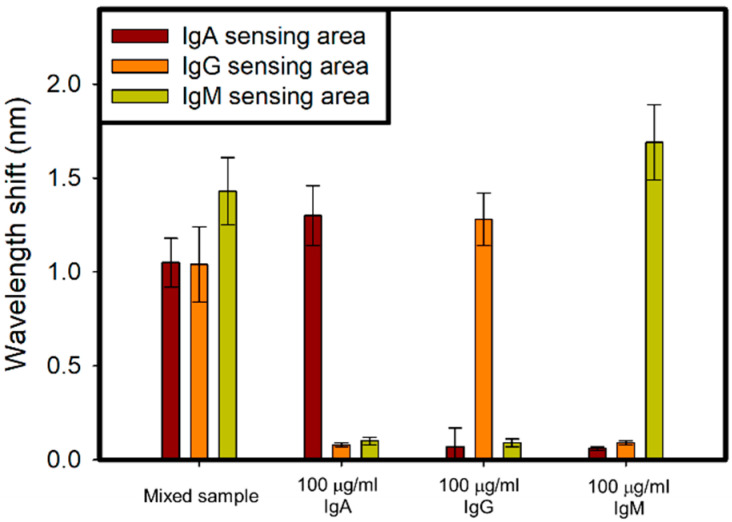
Sample test including 100 μg/mL IgA, IgG, IgM, and mixing sample of IgA, IgG, and IgM with raito of 1:1:1.

To prove the concept, 100 μg/mL IgA, IgG, IgM, and their mixed sample with raito of 1:1:1 were flowed into device. With 100 μg/mL IgA, IgG and IgM flowing through, only their corresponding sensing area hve wavelength shift, respectively. The 100 μg/mL IgA, IgG, IgM mixed sample with raito of 1:1:1, corresponding to concentration of 33.33 μg/mL for each analyte, was flowed into device, and all of three sensing area showed resonant wavelength red-shift in Figure 7.

The detection limit in this SPR sensor is 1 μg/mL, however, several efforts using nanoslit SPR sensors showed the detection limit at the level of several hundreds ng/mL. The reason may be that the sensing area were reduced from several millimeter × millimeter to 150-µm × 150-µm to achieve multiplex and parallel detection in microfluidic channel. When the sensing area become smaller, the sensing variaiton will increase. 

## 4. Conclusions

In summary, a nanoplasmonic sensing array integrated with a parallel microchannel was demonstrated for label-free multiplex detection. The nanostructure on PC film can be massively produced by nanoimprinting. The nanoplasmonic sensing array was fabricated through nanoimprinting and gold deposition which generated Fano-type resonant spectra. The sensitivity of the sensing chip was also evaluated. The sensing chip could also be bound with a PDMS-based microfluidic channel after surface APTES and oxygen plasma treatments. With certain surface modifications, the anti-IgG, anti-IgA, and anti-IgM could be separately conjugated to each surface of the sensing region which could be used to detect IgG, IgA, and IgM in test samples. When IgG, IgA, and IgM were conjugated onto the corresponding sensing regions, the refractive index changed, resulting in red-shifts of the resonant spectra. By analyzing increases of peak values of resonant spectra, concentrations of the targets could be estimated. The results indicated that the developed nanoplasmonic sensing array integrated with parallel microfluidics can be used for multiplex detection. 

## Data Availability

Not applicable.

## References

[1] Yao Y., Huang X.-H., Zhang B.-Y., Zhang Z., Hou D., Zhou Z.-K. (2019). Facile fabrication of high sensitivity cellulose nanocrystals based QCM humidity sensors with asymmetric electrode structure. Sensors Actuators B Chem..

[2] Temel F. (2020). One novel calix [4] arene based QCM sensor for sensitive, selective and high performance-sensing of formaldehyde at room temperature. Talanta.

[3] Lim H.J., Saha T., Tey B.T., Tan W.S., Ooi C.W. (2020). Quartz crystal microbalance-based biosensors as rapid diagnostic devices for infectious diseases. Biosens. Bioelectron..

[4] Dutta P., Lu Y.-J., Hsieh H.-Y., Lee T.-Y., Lee Y.-T., Cheng C.-M., Fan Y.-J. (2021). Detection of *Candida albicans* Using a Manufactured Electrochemical Sensor. Micromachines.

[5] Fan Y.-J., Hsu Y.-C., Gu B.-C., Wu C.-C. (2020). Voltammetric measurement of Escherichia coli concentration through p-APG hydrolysis by endogenous β-galactosidase. Microchem. J..

[6] Teengam P., Siangproh W., Tontisirin S., Jiraseree-Amornkun A., Chuaypen N., Tangkijvanich P., Henry C.S., Ngamrojanavanich N., Chailapakul O. (2020). NFC-enabling smartphone-based portable amperometric immunosensor for hepatitis B virus detection. Sensors Actuators B Chem..

[7] Yang C.-H., Chen C.-W., Lin Y.-K., Yeh Y.-C., Hsu C.-C., Fan Y.-J., Yu I.-S., Chen J.-Z. (2017). Atmospheric-Pressure Plasma Jet Processed Carbon-Based Electrochemical Sensor Integrated with a 3D-Printed Microfluidic Channel. J. Electrochem. Soc..

[8] Chen J., Guo H., Wu Z., Xu G., Zi Y., Hu C., Wang Z.L. (2019). Actuation and sensor integrated self-powered cantilever system based on TENG technology. Nano Energy.

[9] Park C., Kang J., Baek I., You J., Jang K., Na S. (2019). Highly sensitive and selective detection of single-nucleotide polymorphisms using gold nanoparticle MutS enzymes and a micro cantilever resonator. Talanta.

[10] Okan M., Sari E., Duman M. (2017). Molecularly imprinted polymer based micromechanical cantilever sensor system for the selective determination of ciprofloxacin. Biosens. Bioelectron..

[11] Rashed M.Z., Kopechek J.A., Priddy M.C., Hamorsky K.T., Palmer K.E., Mittal N., Valdez J., Flynn J., Williams S.J. (2021). Rapid detection of SARS-CoV-2 antibodies using electrochemical impedance-based detector. Biosens. Bioelectron..

[12] Wang L., Huo X., Qi W., Xia Z., Li Y., Lin J. (2020). Rapid and sensitive detection of Salmonella Typhimurium using nickel nanowire bridge for electrochemical impedance amplification. Talanta.

[13] Park J., Lee W., Kim I., Kim M., Jo S., Kim W., Park H., Lee G., Choi W., Yoon D.S. (2019). Ultrasensitive detection of fibrinogen using erythrocyte membrane-draped electrochemical impedance biosensor. Sensors Actuators B Chem..

[14] Lin C.-Y., Nguyen U.T.N., Hsieh H.-Y., Tahara H., Chang Y.-S., Wang B.-Y., Gu B.-C., Dai Y.-H., Wu C.-C., Tsai I.-J. (2021). Peptide-based electrochemical sensor with nanogold enhancement for detecting rheumatoid arthritis. Talanta.

[15] Fan Y.-J., Huang M.-Z., Hsiao Y.-C., Huang Y.-W., Deng C.-Z., Yeh C., Husain R.A., Lin Z.-H. (2020). Enhancing the sensitivity of portable biosensors based on self-powered ion concentration polarization and electrical kinetic trapping. Nano Energy.

[16] Deng C.-Z., Fan Y.-J., Chung P.-S., Sheen H.-J. (2018). A Novel Thermal Bubble Valve Integrated Nanofluidic Preconcentrator for Highly Sensitive Biomarker Detection. ACS Sensors.

[17] Fan Y.-J., Deng C.-Z., Chung P.-S., Tian W.-C., Sheen H.-J. (2018). A high sensitivity bead-based immunoassay with nanofluidic preconcentration for biomarker detection. Sensors Actuators B Chem..

[18] Wang S.-H., Lo S.-C., Tung Y.-J., Kuo C.-W., Tai Y.-H., Hsieh S.-Y., Lee K.-L., Hsiao S.-R., Sheen J.-F., Hsu J.-C. (2020). Multichannel nanoplasmonic platform for imidacloprid and fipronil residues rapid screen detection. Biosens. Bioelectron..

[19] Lee K.-L., Hou H.-S., Cheng J.-Y., Wei P.-K. (2020). High-Throughput and Dynamic Study of Drug and Cell Interactions Using Contrast Images in Aluminum-Based Nanoslit Arrays. Anal. Chem..

[20] Chuang C.-S., Wu C.-Y., Juan P.-H., Hou N.-C., Fan Y.-J., Wei P.-K., Sheen H.-J. (2019). LMP1 gene detection using a capped gold nanowire array surface plasmon resonance sensor in a microfluidic chip. Analyst.

[21] Fan Y.-J., Sheen H.-J., Hsu C.-J., Liu C.-P., Lin S., Wu K.-C. (2009). A quantitative immunosensing technique based on the measurement of nanobeads’ Brownian motion. Biosens. Bioelectron..

[22] Fan Y.-J., Sheen H.-J., Liu Y.-H., Tsai J.-F., Wu T.-H., Wu K.-C., Lin S. (2010). Detection of C-Reactive Protein in Evanescent Wave Field Using Microparticle-Tracking Velocimetry. Langmuir.

[23] Chuang C.-S., Deng C.-Z., Fang Y.-F., Jiang H.-R., Tseng P.-W., Sheen H.-J., Fan Y.-J. (2019). A Smartphone-based Diffusometric Immunoassay for Detecting C-Reactive Protein. Sci. Rep..

[24] Shankaran D.R., Gobi K.V., Miura N. (2007). Recent advancements in surface plasmon resonance immunosensors for detection of small molecules of biomedical, food and environmental interest. Sensors Actuators B Chem..

[25] Mahmoudpour M., Dolatabadi J.E.N., Torbati M., Tazehkand A.P., Homayouni-Rad A., de la Guardia M. (2019). Nanomaterials and new biorecognition molecules based surface plasmon resonance biosensors for mycotoxin detection. Biosens. Bioelectron..

[26] Zhao J., Zhang X., Yonzon C.R., Haes A.J., Van Duyne R.P. (2006). Localized surface plasmon resonance biosensors. Nanomedicine.

[27] Zhou J., Qi Q., Wang C., Qian Y., Liu G., Wang Y., Fu L. (2019). Surface plasmon resonance (SPR) biosensors for food allergen detection in food matrices. Biosens. Bioelectron..

[28] Mahmoudpour M., Dolatabadi J.E.N., Torbati M., Homayouni-Rad A. (2018). Nanomaterials based surface plasmon resonance signal enhancement for detection of environmental pollutions. Biosens. Bioelectron..

[29] Lu M., Zhu H., Bazuin C.G., Peng W., Masson J.-F. (2019). Polymer-Templated Gold Nanoparticles on Optical Fibers for Enhanced-Sensitivity Localized Surface Plasmon Resonance Biosensors. ACS Sensors.

[30] Culver H.R., Wechsler M.E., Peppas N.A. (2018). Label-Free Detection of Tear Biomarkers Using Hydrogel-Coated Gold Nanoshells in a Localized Surface Plasmon Resonance-Based Biosensor. ACS Nano.

[31] Petryayeva E., Krull U.J. (2011). Localized surface plasmon resonance: Nanostructures, bioassays and biosensing—A review. Anal. Chim. Acta.

[32] Fano U. (1941). The Theory of Anomalous Diffraction Gratings and of Quasi-Stationary Waves on Metallic Surfaces (Sommerfeld’s Waves). J. Opt. Soc. Am..

[33] Miroshnichenko A., Flach S., Kivshar Y.S. (2010). Fano resonances in nanoscale structures. Rev. Mod. Phys..

[34] Luk’Yanchuk B., Zheludev N., Maier S., Halas N., Nordlander P., Giessen H., Chong C.T. (2010). The Fano resonance in plasmonic nanostructures and metamaterials. Nat. Mater..

[35] Lee K.-L., Huang J.-B., Chang J.-W., Wu S.-H., Wei P.-K. (2015). Ultrasensitive Biosensors Using Enhanced Fano Resonances in Capped Gold Nanoslit Arrays. Sci. Rep..

[36] Guzniczak E., Otto O., Whyte G., Willoughby N., Jimenez M., Bridle H.L. (2020). Deformability-induced lift force in spiral microchannels for cell separation. Lab. Chip.

[37] Lin S., Zhi X., Chen D., Xia F., Shen Y., Niu J., Huang S., Song J., Miao J., Cui D. (2019). A flyover style microfluidic chip for highly purified magnetic cell separation. Biosens. Bioelectron..

[38] Luo T., Fan L., Zeng Y., Liu Y., Chen S., Tan Q., Lam R.H.W., Sun D. (2018). A simplified sheathless cell separation approach using combined gravitational-sedimentation-based prefocusing and dielectrophoretic separation. Lab. Chip.

[39] Fan Y.-J., Hsieh H.-Y., Tsai S.-F., Wu C.-H., Lee C.-M., Liu Y.-T., Lu C.-H., Chang S.-W., Chen B.-C. (2021). Microfluidic channel integrated with a lattice lightsheet microscopic system for continuous cell imaging. Lab. Chip.

[40] Fan Y.-J., Hsiao Y.-C., Weng Y.-L., Chen Y.-H., Chiou P.-Y., Sheen H.-J. (2020). Development of a parallel three-dimensional microfluidic device for high-throughput cytometry. Sensors Actuators B Chem..

[41] Daguerre H., Solsona M., Cottet J., Gauthier M., Renaud P., Bolopion A. (2020). Positional dependence of particles and cells in microfluidic electrical impedance flow cytometry: Origin, challenges and opportunities. Lab. Chip.

[42] Mutafopulos K., Spink P., Lofstrom C.D., Lu P.J., Lu H., Sharpe J.C., Franke T., Weitz D.A. (2019). Traveling surface acoustic wave (TSAW) microfluidic fluorescence activated cell sorter (μFACS). Lab. Chip.

[43] Zhao Y., Zhang W., Zhao Y., Campbell R.E., Harrison D.J. (2019). A single-phase flow microfluidic cell sorter for multiparameter screening to assist the directed evolution of Ca2+ sensors. Lab. Chip.

[44] Zhao J., You Z. (2018). Spark-generated microbubble cell sorter for microfluidic flow cytometry. Cytom. Part A.

